# Enhancing Biomethane Production From Lignite by an Anaerobic Polycyclic Aromatic Hydrocarbon Degrading Fungal Flora Enriched From Produced Water

**DOI:** 10.3389/fmicb.2022.899863

**Published:** 2022-05-26

**Authors:** Kaiyi Gong, Yixuan Zhang, Hongguang Guo, Zaixing Huang, Michael Urynowicz, Muhammad Ishtiaq Ali

**Affiliations:** ^1^College of Safety and Emergency Management and Engineering, Taiyuan University of Technology, Taiyuan, China; ^2^Key Lab of In-situ Property-improving Mining of Ministry of Education, Taiyuan University of Technology, Taiyuan, China; ^3^School of Chemical Engineering and Technology, China University of Mining and Technology, Xuzhou, China; ^4^Department of Civil and Architectural Engineering, University of Wyoming, Laramie, WY, United States; ^5^Environmental Microbiology Lab, Department of Microbiology, Quaid-I-Azam University, Islamabad, Pakistan

**Keywords:** microbially enhanced coalbed methane, fungi, polycyclic aromatic hydrocarbons, volatile fatty acids, functional groups

## Abstract

The coal-degrading ability of microorganisms is essential for the formation of biogenic coalbed methane. The ability to degrade the aromatic compound of coal is more important because it is perceived as the main refractory component for bioconversion. In this paper, a polycyclic aromatic hydrocarbon (PAH) degrading fungal community (PF) was enriched from produced water using phenanthrene as sole carbon source. The goal was to improve both the microbial structure of the methanogenic microflora and its coal-degrading ability. Two strategies were pursued. The first used coal pretreatment with PF (PP), followed by methane production by methanogenic microflora; the second used methane production directly from coal by mixed culture of PF and methanogenic microflora (PM). The results showed that methane productions of PP and PM increased by 29.40 and 39.52%, respectively. After 7 days of cultivation, the fungal community has been altered in PP and PM, especially for *Penicillium* the proportions of which were 67.37 and 89.81% higher than that in methanogenic microflora, respectively. Furthermore, volatile fatty acid accumulations increased by 64.21 and 58.15%, respectively. The ^13^C-NMR results showed that PF addition promoted the transformation of aromatic carbons in coal to carboxyl and carbonyl carbons, which contributed greatly to the production of methane together with oxygen-containing functional groups. These results suggest that methane production can be increased by indigenous PAH-degrading fungi by improving the fermentation of aromatics in coal and the generation of volatile fatty acids. This provided a feasible method for enhancing biomethane generation in the coal seam.

## Introduction

Approximately 20% of the natural gas in the world, including coalbed methane (CBM), is formed by the biogenic pathway (Ritter et al., 2015). Biogenic CBM is predominant in certain basins (Strapoć et al., 2011), which leads to increasing interest in the technology of microbially enhanced CBM (MECBM). MECBM has been proposed based on the formation of biogenic CBM, which converts coal into methane by injecting either microorganisms and nutrients or nutrients alone into both the original coal seam and the coal seam after mining (Scott, 1999). Consequently, MECBM can improve the CBM yield and extend the productive lifespans of CBM wells (Ritter et al., 2015).

Coal is a macromolecular geopolymer composed of aromatic, aliphatic, heterocyclic, and lignin-derived compounds (Dariusz et al., 2008; Strapoć et al., 2011). Microorganism-mediated methane generation from coal begins with coal fragmentation, such as hydrolysis of aromatics. This is then followed by step by step fermentation of organic intermediates with the production of methanogenic substrates which is then further utilized by methanogens to generate biomethane. However, the complexity of the coal structure obstructs this process, especially the first step of coal biodegradation which is believed to be the main obstacle and limiting factor for biogenic CBM generation (Park and Liang, 2016). Aromatics are the main organics in coal, but their degradation by microorganisms is more difficult than that of aliphatic hydrocarbon (Wang et al., 2018; Guo et al., 2019). Therefore, for the generation of biogenic CBM, it is beneficial to promote the degradation of aromatic compounds in coal.

At present, the major technologies that can improve coal degradation are chemical and biological pretreatments of coal. Hydrogen peroxide, nitric acid, and potassium permanganate are common reagents for the chemical pretreatment of coal. After pretreating sub-bituminous coal with hydrogen peroxide, the solubilization and bioconversion of coal are improved and methane production increases 10 fold (Chen et al., 2018). Huang et al. (2013) used HNO_3_, catalyzed H_2_O_2_, KMnO_4_, and NaOH to pretreat sub-bituminous coal, and found that HNO_3_ treatment was optimal for coal conversion by fungal enzyme manganese peroxidase. Aerobic microorganisms are usually used for the biological pretreatment of coal. Wang et al. (2017) pretreated lignite with an aerobic bacterial flora acclimated from aerobic sludge, which resulted in an increased methane production of 144.82 μmol/g coal. *Penicillium chrysogenum* was used to degrade lignite in an aerobic environment, and fungal extracts containing nitrogenous, cyclic, and aromatic compounds were utilized by the methanogenic microflora (MM) to obtain higher methane production (Haider et al., 2013). As oxygen is absent in coal seams, the biological pretreatment by aerobic microbes is limited in *in situ* applications. Instead, pretreatment by anaerobic microorganisms is more feasible, especially microorganisms that survive in the coal seam as they already are anaerobic and adapted to the physicochemical environment underground.

Fungi are of great interest in the degradation of aromatics and the bioconversion of coal because of their outstanding capacity for degrading organics with large molecules and complex structures. *Candida, Fusarium, Aspergillus, Penicillium*, and *Pichia* are the most common fungal genera used to remove polycyclic aromatic hydrocarbons (PAHs) such as naphthalene, phenanthrene, dibenzothiophene, and pyrene (Kashyap et al., 2020). Various fungi have been reported to solubilize low-rank coal, especially aromatics present in coal. For example, *Trichoderma, Penicillium*, and *Phanerochaete* have been recognized to solubilize coal and to be involved in the degradation of aromatic compounds (Detman et al., 2018). *Phanerochaete* can degrade PAHs in coal tar and break down lignin polymers (Bogan and Lamar, 1995). *Aspergillus, Candida, Penicillium, Mucor*, and *Paecilomyces* have been isolated from lignite and all of these can use coal as sole carbon source (Ward, 1985). *Rhizopus oryzae* has been separated from low-rank coal in Qasam Khel, Pakistan, and solubilized 36.8% of coal after 9 days of cultivation (Sabar et al., 2019). Moreover, many fungi have been found to have a strong ability to degrade PAHs under microaerobic and very-low-oxygen conditions; prominent examples are *Aspergillus* sp., *Trichocladium canadense, Fusarium oxysporum, Verticillium* sp., and *Achremonium* sp. (Silva et al., 2009). In coal seams, it has been previously demonstrated that *in situ* fungi play an important role in coal biodegradation and biogenic CBM formation; these also have a higher potential to synergistically generate methane with methanogens than bacteria (Guo et al., 2017). The syntrophic relationship between fungi and hydrogenotrophic methanogens in produced water was detected in the laboratory during methane production from anthracite (Guo et al., 2020b). Thus, using aromatics-degrading fungal communities cultivated from CBM field would be a good way to promote methane production from coal.

In this paper, a PAH-degrading fungal flora (PF) was enriched from produced water using phenanthrene as sole carbon source. Two strategies were explored for improving coal degradation and methane generation. One uses MM composed of fungi and methanogens for methane generation after coal pretreatment with PF. The other generates methane from coal by mixed culture of PF and MM. The mechanism of methane enhancement was also assessed by determining changes of the microbial community, intermediates, and functional groups of coal during methane production. To the best of our knowledge, this is the first paper presenting the use of indigenous anaerobic PF to enhance biogenic CBM generation.

## Materials and Methods

### Sample Collection

Produced water samples were collected from active CBM wells in the southeast of Qinshui Basin, Shanxi, China. They were collected into 2 L sterile bottles, carefully maintaining all air in the headspaces clean. After sampling, bottles were tightly sealed and transported to the laboratory on ice as quickly as possible. Lignite was sampled from the Baorixile coal mine in Inner Mongolia, China, and pulverized to <200 mesh in the laboratory. Proximate and ultimate analyses of coal samples were performed according to GB/T 212-2008 (Nie et al., 2015). The results showed that the air-dry basis moisture, ash yield, volatile matter, and fixed carbon were 1.95, 10.65, 33.47, and 53.93%, respectively. The contents of carbon, hydrogen, oxygen, nitrogen, and sulfur were 65.20, 3.47, 17.53, 0.79, and 0.38%, respectively.

### Enrichment of Polycyclic Aromatic Hydrocarbon Degrading Fungal Flora and Methanogenic Microflora

Produced water was the source of microorganisms. Lignite and phenanthrene were used as sole carbon sources to enrich MM and PF, respectively. Totals of 0.2 mM streptomycin and 0.1 mM ampicillin solutions were added to inhibit bacterial activity in MM and PF (Guo et al., 2017, 2020b). Anaerobic medium (1 L) was composed of 100 ml basic medium, 30 mL trace element solution, 30 mL vitamin solution, and 100 mL cysteine (15%)—Na_2_S (15%). The basic medium contained KCl (33.5 g/L), CaCl_2_·2H_2_O (14 g/L), MgCl_2_·6H_2_O (275 g/L), NH_4_Cl (25 g/L), MgSO_4_·7H_2_O (345 g/L), NaCl (1100 g/L), and K_2_HPO_4_·3H_2_O (14 g/L). The trace element solution contained FeCl_2_·4H_2_O (1500 mg/L), AlK(SO_4_)_2_ (10 mg/L), ZnCl_2_ (70 mg/L), NiCl_2_·6H_2_O (24 mg/L), MnCl_2_·4H_2_O (100 mg/L), NaMoO_4_ (6 mg/L), CuCl_2_ (2 mg/L), H_3_BO_3_ (36 mg/L), CoCl_2_·6H_2_O (190 mg/L), and 0.25% HCl. The vitamin solution contained biotin (2 mg/L), folic acid (2 mg/L), pyroxidine HCl (10 mg/L), thiamine HCl (5 mg/L), riboflavin (5 mg/L), nicotinic acid (5 mg/L), lipoic acid (5 mg/L), aminobenzoic acid (5 mg/L), and vitamin B12 (1 mg/L). A total of 27 mL of produced water sample was added in an autoclaved 100 mL serum bottle with 3 mL anaerobic medium and 1 g of pulverized coal. The headspace was filled with N_2_ gas at 1 atm. All bottles were incubated in the dark without shaking at 35°C. Every 4 to 5 weeks, 10% of the enrichment was transferred to a fresh medium containing 1 g of coal sample and cultured under the same conditions. The methane concentration in the headspace of serum bottles was measured every week using a gas chromatograph.

Mineral salt medium (MSM) was used for PF enrichment, which was composed of KH_2_PO_4_ (15.2 g/L), CaCl_2_ (0.5 g/L), MgSO_4_·7H_2_O (2 g/L), (NH_4_)_2_SO_4_ (5 g/L), and Na_2_HPO_4_ (24.4 g/L). A total of 27 mL of produced water sample was added to an autoclaved 100 mL serum bottle with 3 mL MSM and 15 mg of phenanthrene. The culture condition and transferring procedures were the same as those for MM enrichment. The amount of the residual phenanthrene at the end of cultivation was determined to calculate the degradation rate.

### Determination of Phenanthrene

The phenanthrene in the medium cultivated with PF was isolated by liquid/liquid extraction with 30 mL pesticide-grade dichloromethane at pH 3.0, pH 7.0, and pH 11.0. Extraction at each pH was performed three times. NaOH and HCl were used to adjust the pH of solutions after NaCl saturation. The mixture of extracts at different pH levels was dried with an excess of anhydrous sodium sulfate. Then, it was concentrated to 1 mL by roto evaporation followed by a gentle stream of nitrogen, which was used for gas chromatography-mass spectrometry (GC-MS) analysis. The external standard method was employed to quantify phenanthrene in culture (Yun and Minyan, 2014). Dichloromethane was also used to prepare phenanthrene standard solutions. A calibration curve was made from 1,500, 1,200, 900, 600, and 300 mg/L of standard phenanthrene solutions. Standard solution samples (1 mL) of each concentration were analyzed by GC-MS. The concentrations of residual phenanthrene in these samples were calculated based on the standard curve (Supplementary Figure 1). Cultivations without microbes were set as controls to determine the recovery of phenanthrene. All experiments were performed in three replicates.

### Methane Production

Two treatments were set up to enhance both coal degradation and methane production. One treatment produced methane from coal by MM after pretreatment with PF (abbreviated PP). The other produced methane from coal by mixed cultivation of PF and MM (abbreviated PM). In PP, 3 mL PF and 1 g coal were added into a 100 mL serum bottle with 27 mL anaerobic medium. After 7 days, 3 mL MM was added to produce methane. In PM, 0.5 mL PF, 2.5 mL of MM, and 1 g coal were added into a 100 mL serum bottle with 27 mL anaerobic medium to produce methane directly. Cultivations with anaerobic medium and coal were set as controls. The headspaces of bottles were filled with N_2_ gas. The bottles were incubated without shaking at 35°C. A control was performed where coal was soaked with anaerobic medium to determine the effect of the medium on the organics released from coal. All experiments were performed in three replicates. The concentration of methane production was determined every week.

During methane production, both the fermentation broth and residual coal were collected and changes in microbial community, dissolved organic matter, and coal structure were analyzed. Residual coal was separated by a glass fiber filter (0.7 μm, GF/F, Whatman, Little Chalfont, UK). Residual coal on the fiber filter was removed and dried at 105°C to a constant weight for ^13^C-nuclear magnetic resonance (^13^C-NMR) analysis. The organic matters in the filtrate were extracted by the same liquid-liquid extraction method as mentioned above for the determination of phenanthrene, and were analyzed by GC-MS.

### DNA Extraction and Miseq

The fermentation broth was filtered through a 0.22 μm membrane filter (Millipore, USA) to collect microorganisms for DNA extraction. The genomic DNA of each sample was extracted using the UltraClean Soil DNA Isolation Kit (Mobio, USA) according to the manufacturer's instructions. The archaea-specific primer sets Arch519F-Arch915R (Yu et al., 2020) and the fungi-specific primer sets ITS1F-ITS2R (Mueller et al., 2014) were used to amplify the DNA of archaeal and fungal communities, respectively. The PCR reactions were performed as follows: 95°C for 5 min, 27 cycles at 95°C for 30 s, 55°C for 30 s, 72°C for 45 s, and finally, 72°C for 10 min. The obtained PCR products were purified with the AxyPrep DNA gel recovery kit (Axygen Biosciences, USA). Sequencing was performed on an Illumina Miseq platform.

After sequencing, Trimmomatic soft was used to assess the quality of all sequence reads according to the following thresholds: sequence reads with a length of >50 bp, no ambiguous bases in the entire sequence, and no mismatches in the primer sequence. Any reads with poor quality and primer dimers were removed (Bolger et al., 2014). Usearch verson 7.1 (http://drive5.com/uparse/) was used to assign operational taxonomic units (OTUs) below 97% similarity. A representative sequence from each OTU was selected and assigned to taxonomic rank by comparison against the Silva database (Pruesse et al., 2007) and the Unite fungal database (Kõljalg et al., 2013). Mothur version 1.30.1 was used to calculate diversity and richness estimators such as Chao1, Shannon, Simpson, and Coverage (Schloss et al., 2009). Rarefaction analysis was also performed by Mothur. The 16S rRNA gene and ITS gene sequences derived from Miseq were deposited in the NCBI Sequence Read Archive under accession number PRJNA754200.

### Gas Chromatography-Mass Spectrometry Analysis

The phenanthrene in the culture and the organics in the filtrate were quantitatively analyzed by GC-MS (Agilent, 7890B-5977B, USA) equipped with an HP-INNOWax column (30 m × 0.25 mm × 0.25 μm). A total of 1 μL extracted sample solution was injected automatically. Helium was used as carrier gas at a flow rate of 0.75 mL/min. The following temperature program was adopted: an initial 60°C for 3 min, which was then gradually increased to 150°C at 20°C/min, finally increased to 230°C at 5°C/min, which was maintained for 5 min. Quantitative analysis of phenanthrene was based on the mode of selected ion monitoring. Qualitative ion was 178 m/z, and quantitative ions were 176, 178, and 178 m/z, respectively (He et al., 2019). Organic matter in the fermentation broth was monitored in scanning mode. The compounds obtained from GC-MS were identified using the database of the National Institute Standard and Technology (NIST 14 L). The relative abundance of organic matter was calculated by peak area normalization (Fiori et al., 2010).

### ^13^C-Nuclear Magnetic Resonance Analysis

^13^C-NMR was employed to determine the changes of functional groups in coal by microorganisms, performed on an Agilent 600 DD2 spectrometer at a resonance frequency of 150.15 MHz. ^13^C-NMR spectra were recorded by spinning at 5 kHz with a 4 mm probe at room temperature. The delay time was 5 s and 2,048 scans were conducted. The chemical shifts regions of ^13^C-NMR spectra for each functional group in coal were analyzed by MestReNova software.

## Results and Discussion

### Characteristics of Methanogenic Microflora

**Figure 1:**
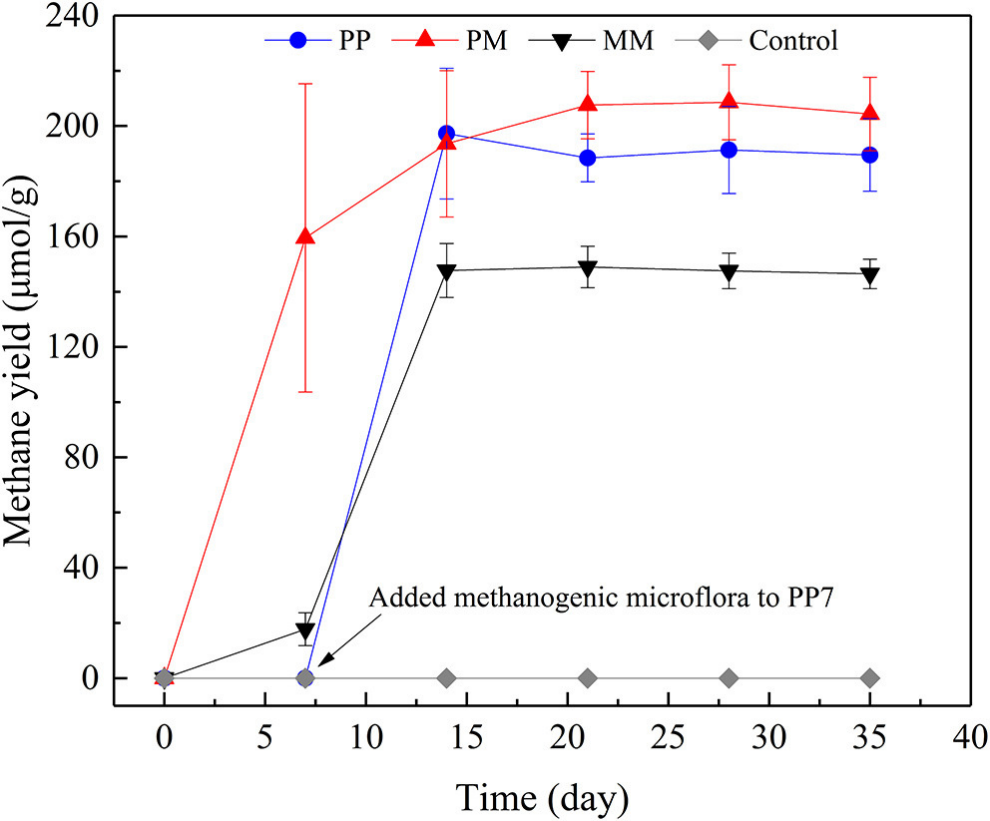
Methane productions from raw coal by methanogenic microflora (MM), from coal treated by PAHs-degrading fungal flora (PF) for 7 days (PP), from raw coal by mixed culture of PF and MM at a ratio of 1:5 (PM), and from raw coal without methanogenic microflora (Control).

After about 20 transfers, MM was successfully enriched with a maximum methane yield of 146.45 μmol/g coal (Figure 1). Slow, fast, and stable stages of methane generation from coal were found which had also been reported in another study (Wang et al., 2017). Methane generation in the first 7 days only increased to 17.73 μmol/g coal and the methanogenic rate was 2.53 μmol/day. From days 7 to 14, the methane yield increased rapidly to 147.68 μmol/g coal and the methanogenic rate was as high as 18.56 μmol/day. Methane production changed a little after 14 days of cultivation which might be due to the toxic conditions caused by the accumulation of inhibitory intermediates released from coal (Jones et al., 2010). A further possible reason may be the lack of nutrients (Zhang and Liang, 2017) which inhibited the activity of methanogens.

**Figure 2:**
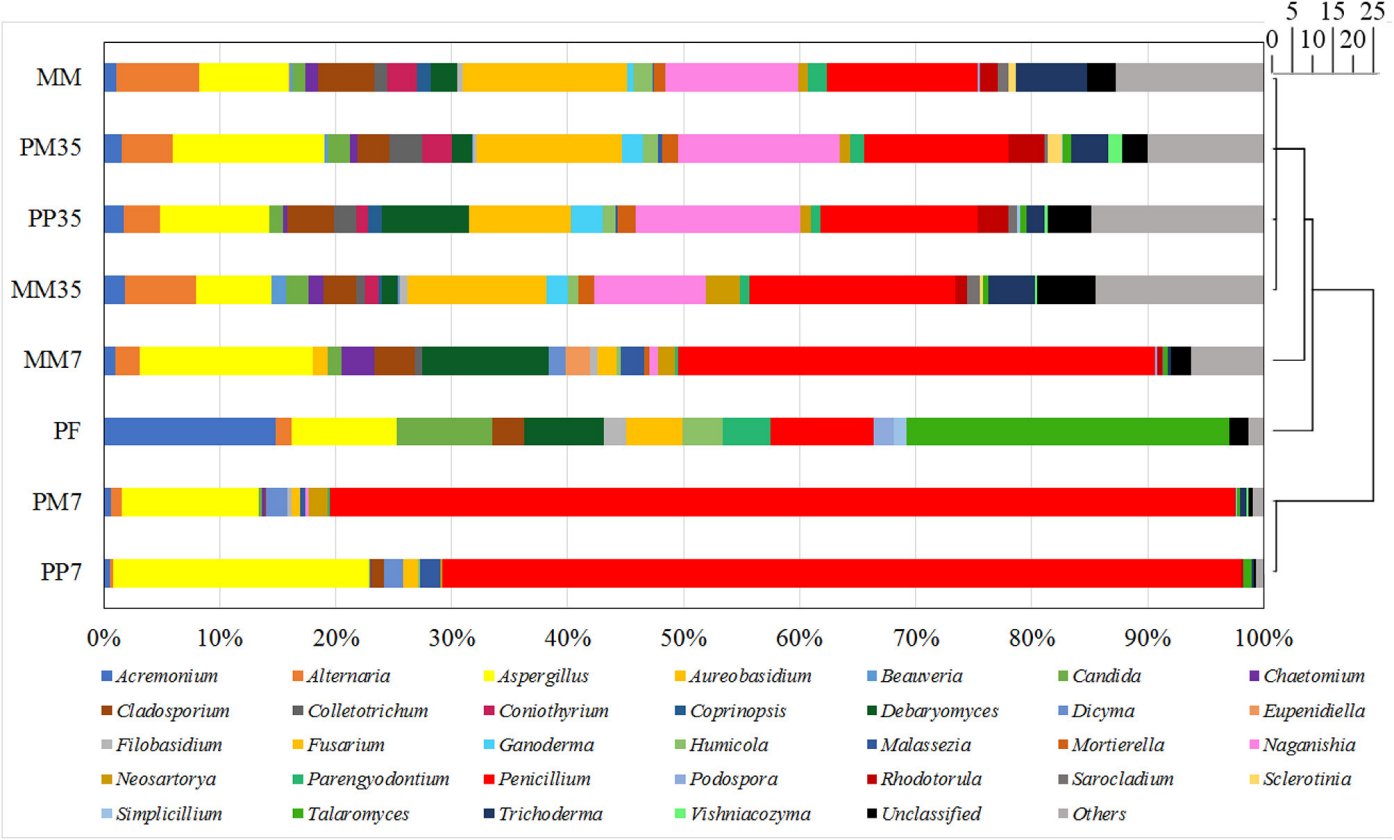
Fungal community compositions at genus level. MM represents the cultivations with raw coal and methanogenic microflora. PP represents the cultivations with PF pretreated coal and methanogenic microflora. PM represents the cultivations with raw coal and mixed culture of PF and methanogenic microflora. The 7 and 35 are the sampling time. The genera accounted for <1% of sequence reads were grouped into others in each sample. The scale bar represents the phylogenetic distance between samples.

**Figure 3:**
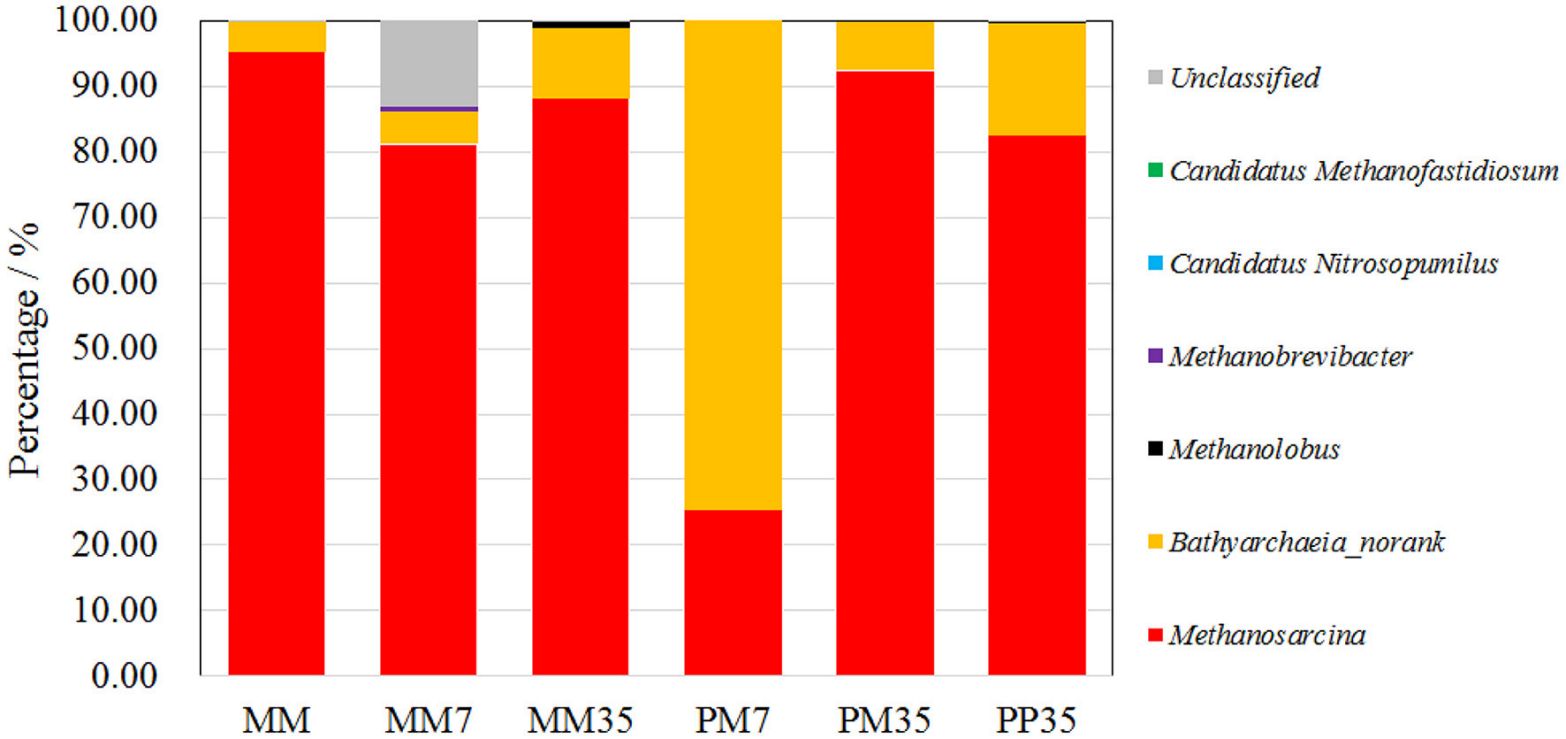
The composition of archaeal community at genus level. PP represents the cultivations with PF pretreated coal and methanogenic microflora. PM represents the cultivations with raw coal and mixed culture of PF and methanogenic microflora. MM represent the methanogenic microflora after enrichment. The 7 and 35 respectively represent the incubation time.

The number of sequence reads and OTUs, as well as the estimators of Shannon, Simpson, and coverage are shown in Supplementary Table 1. A total of 40,560 fungal sequence reads and 29,827 archaeal sequence reads were grouped into 198 and 97 OTUs with Shannon estimators of 3.99 and 0.82, respectively. The coverages were both above 0.999. The composition of the fungal community in MM is shown in Figure 2 and the details are provided in Supplementary Table 2. The dominant fungi included *Fusarium* (14.24%), *Penicillium* (13.04%), *Naganishia* (11.42%), *Aspergillus* (7.74%), *Alternaria* (7.10%), and *Trichoderma* (6.15%). Most of these fungi are related to coal degradation and the formation of methanogenic intermediates. For example, *Penicillium, Aspergillus*, and *Trichoderma* all had an outstanding capacity of solubilizing low-rank coal (Ward, 1985; Zafra et al., 2015; Detman et al., 2018). *Fusarium* is one of the main fungi in the culture of coal degradation that produces methane (Guo et al., 2020a). *Alternaria* can provide a more favorable environment for methanogens by reducing sulfur and ash from coal (Guo et al., 2020a). The composition of the archaeal community is shown in Figure 3 and Supplementary Table 3. *Methanosarcina*, which can utilize multiple substrates to produce methane including H_2_/CO_2_, acetate, methanol, dimethyl sulfide, and methylamines (He et al., 2020), accounted for 95.17% of all sequence reads. The diverse methanogenic pathways and excellent environmental adaptability of *Methanosarcina* are conducive to generate methane (Vrieze et al., 2012).

### Characteristics of Polycyclic Aromatic Hydrocarbon Degrading Fungal Flora

PF was obtained by enrichment culture for tens of times and its ability to degrade phenanthrene was detected by GC-MS. The recovery of phenanthrene in the control group was 95.75 ± 3.56%. After 14 days of culture, the degradation rate of phenanthrene by PF was 43.99 ± 9.36%, suggesting that PF had a good ability to degrade phenanthrene.

After Miseq, 38,377 fungal sequence reads with 21 OTUs were obtained (Supplementary Table 1). The Shannon estimator of 2.43 and Chao1 of 21 were both significantly lower than those of MM. The fungal community structure of PF is shown in Figure 2 and the details for the proportions of fungi are presented in Supplementary Table 2. The clustering results showed that the fungal community in PF was different from that in MM. Although most fungal genera were simultaneously detected in both samples, the proportion of each genus and the dominant genera varied. The dominant fungal genera containing more than 5.00% of sequence reads in PF were *Talaromyces* (27.87%), *Acremonium* (14.79%), *Aspergillus* (9.10%), *Penicillium* (8.89%), *Candida* (8.20%), and *Debaryomyces* (6.93%). All of these were reported to possess excellent PAH degradation ability. Members of *Talaromyces* and *Aspergillus* have been isolated from soil contaminated by crude oil and can degrade phenanthrene and pyrene (Reyes-César et al., 2014). *Talaromyces* was reported to degrade three-ring PAHs and effectively remove phenanthrene (Gao et al., 2019). *Aspergillus* has been reported to metabolize pyrene, benzo[a]pyrene, and phenanthrene (Passarini et al., 2011; Reyes-César et al., 2014). *Aspergillus* even has an excellent ability to degrade PAHs with 2–7 rings under microaerobic and very-low-oxygen conditions (Silva et al., 2009). *Acremonium* sp. is a facultatively anaerobic fungus (Kurakov et al., 2008), which has an excellent ability to remove naphthalene, fluorene, phenanthrene, anthracene, and fluoranthene (Ma et al., 2014). Both *Acremonium* and *Talaromyces* have a high tolerance to soil contaminated with high-level crude oil and a good PAH degradation ability (Zafra et al., 2014; Ma et al., 2015), which may be the reason for their high proportion in PF. *Penicillium* has been detected in the produced water of CBM well in China's Southern Qinshui Basin (Guo et al., 2017), and has been found to both degrade and solubilize different coal varieties (Ghani et al., 2015). *Candida* has been isolated from activated sludge under anaerobic conditions (Wen et al., 2006) and can biodegrade phenanthrene (Kashyap et al., 2020). *Debaryomyces* was used to remove Benzo[ghi]perylene and phenols (Mojiri et al., 2019) and can also grow under anaerobic conditions (Campbell and Msongo, 1991).

### Enhancement of Methane Production by Polycyclic Aromatic Hydrocarbon Degrading Fungal Flora

The enhancement of methane production by PF was carried out in two ways. One used the pretreatment of coal by PF for 7 days. In the other, PF was mixed directly with MM. The methane yields of both treatments are shown in Figure 1. After incubated for 35 days, the methane yields of the cultivations PP and PM were 189.51 and 204.33 μmol/g coal, respectively, which represent increases by 29.40 and 39.52% compared with MM. Methane was not observed in the control group. In PM, the highest methanogenic rate of 22.79 μmol/day was observed from 0 to 7 day. In PP, no methane production was detected over the first 7 days, as no methanogenic microflora was added. After the addition of methanogenic microflora, the methanogenic rate reached 28.17 μmol/day from days 7 to 14, which was the highest level among all treatments. Compared with MM, the methanogenic rates of both PP and PM increased by 51.78 and 22.79%, respectively. These results indicate that the methane production could be effectively improved by PF through both ways, making them promising choices for increasing methane production. The procedure of PP is complex and the process of pretreatment is time-consuming; the procedure of PM is more convenient for on-site operation.

When coal was pretreated by PF, the biodegradation of aromatic compounds in coal is mostly completed within the first 7 days. The methanogenic microflora added later could directly use the metabolites released from coal by PF, thus shortening the step of coal degradation by MM and greatly promoting the methanogenic rate. When PF was mixed with the methanogenic microflora, the biodegradation of aromatic compounds in coal might also be enhanced, causing significantly increases of methanogenic rate and methane production in PM.

### Effect of Polycyclic Aromatic Hydrocarbon Degrading Fungal Flora on Microbial Communities During Methane Production

The diversities and compositions of fungal communities during methane production are summarized in Supplementary Tables 1, 2, and Figure 2. Compared with MM, the fungal diversities decreased initially in MM7 and then increased further in MM35 as the culture progressed (7 and 35 are the sampling time). These increases reflect the process of microbial adaptation to the new environment. The changes of fungal diversities in PP and PM were similar. The Shannon indexes in both treatments decreased on day 7 and increased from 1.93 to 4 and 1.81 to 3.94 during the later stage, respectively.

According to the cluster analysis shown in Figure 2, although two different strategies were applied, the fungal communities on day 7 in both treatments (PP7 and PM7) were similar which was also demonstrated by the fungal composition. *Aspergillus* and *Penicillium* were abundant fungal genera in both PP7 (22.08 and 68.79%, respectively) and PM7 (11.84 and 78.01%, respectively), while they were 14.93 and 41.10% in MM7. The proportions of *Penicillium* in PP7 and PM7 increased notable by 67.37 and 89.81%, respectively. The dominance of *Aspergillus* and *Penicillium* is beneficial to the biodegradation of lignite and the formation of methane because they are excellent fungi for solubilizing low-rank coal (Ward, 1985; Detman et al., 2018). These results showed that PF altered the fungal community and governed microbial changes in the first 7 days.

The microbial communities of PF changed clearly when they were cultivated with coal. The proportions of dominant fungi in PF such as *Acremonium, Candida*, and *Talaromyces* were all <5% in PP7. This might be because of the diverse organics introduced by coal and the unique structure of coal. Coal-derived organics not only include aromatics, but also a mixture of aliphatic, heterocyclic, and lignin-derived compounds (Strapoć et al., 2011). Furthermore, coal features rambling pores and a fracture structure, irregular chemical composition, and toxic organic matters (Raudsepp et al., 2017). These complex contents and structures probably led to the gradual elimination of certain PAH-degrading fungi, while fungi such as *Aspergillus* and *Penicillium* [which have the potentiality to degrade coal and perform diverse metabolic functions including cellulose (Fouda et al., 2019) and aromatics fermentation (Ali, 2012)] increased significantly. Moreover, *Aspergillus* and *Penicillium* also have good adaptability to extreme environments such as high temperature, acidity/alkalinity, salinity, pressure, and radiation (Xu et al., 2014). This suggests that they propagated largely in the early stage and improved the degradation of coal to provide a suitable environment and intermediate metabolites for methanogens and other fungi.

The fungal community compositions of PP35, PM35, and MM35 were similar in that *Aspergillus, Fusarium, Naganishia*, and *Penicillium* were the dominant fungal genera. At the end of methanogenesis, the abundances of *Aspergillus* and *Penicillium* suddenly decreased in cultivations PP, PM, and MM, which further corroborated that *Aspergillus* and *Penicillium* were more active in the early stage. Considering that PF and MM were enriched from the same produced water sample and that fungal communities in different treatments overlapped, PF addition would not negatively influence MM and might be assimilated gradually by MM during cultivation. The essential role of PF would be the promotion of the breakage and degradation of coal, especially the aromatics in coal, in the early stage. This was mainly achieved by coal-degrading fungi such as *Aspergillus* and *Penicillium*, leading to increased methane production.

The compositions of the archaeal community are shown in Figure 3 and the details are summarized in Supplementary Table 3. *Methanosarcina* and *Bathyarchaeia* (no rank) dominated all the samples which together occupied 86.13–99.98% of sequence reads. Specifically, *Methanosarcina* accounted for 25.46–95.17%, and *Bathyarchaeia* for 4.54–74.53%. *Bathyarchaeia*, a novel methylotrophic methanogen (Lyu et al., 2018), was only dominant in PM7 with 74.53% of sequence reads, but accounted for <17.17% in other samples. Clearly, addition of PF in MM enhanced the growth of *Bathyarchaeia* in the early stage of coal biodegradation, suggesting that the methanogenic pathway was also changed by PF addition. The fermentation of coal by PF was more likely to generate substrates for methylotrophic methanogenesis. The high proportion of *Methanosarcina* yielded more diverse energy sources and better environmental adaptation for the methanogenic microflora. This caused the methanogens to change back to *Methanosarcina* at the end of cultivation.

### Effect of Polycyclic Aromatic Hydrocarbons Degrading Fungal Flora on the Organic Compositions During Methane Production

**Figure 4:**
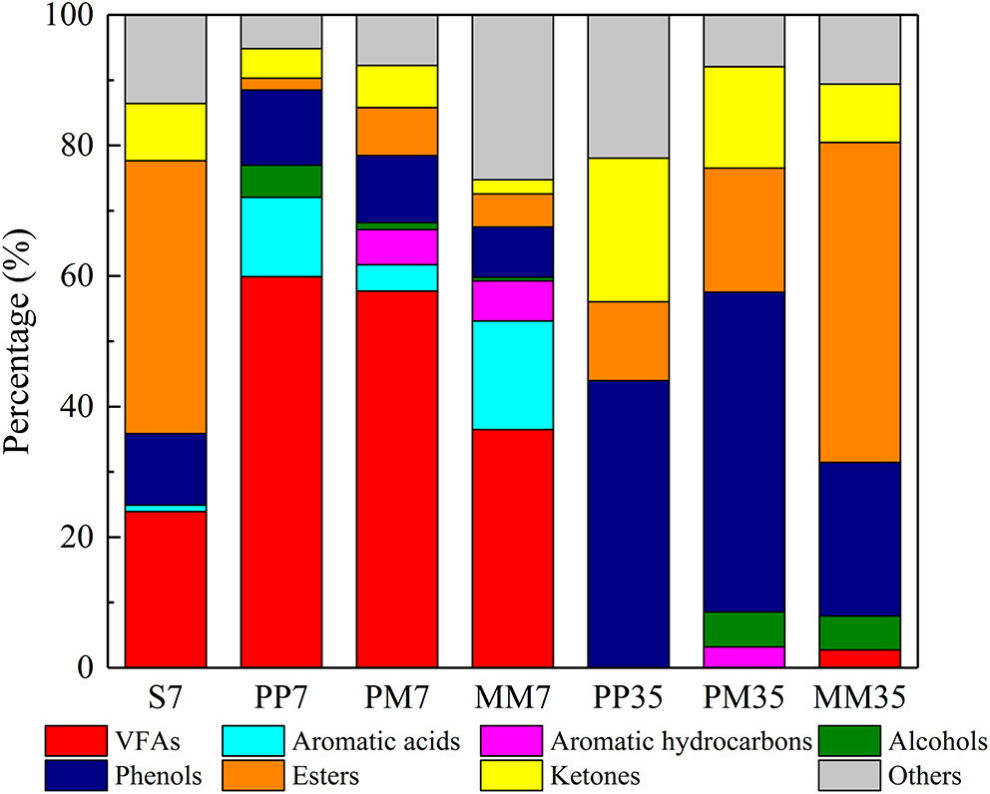
The composition of organic matter in each sample revealed by GC-MS. S represents soaked coal with only anaerobic medium. PP represents the cultivations with PF pretreated coal for 7 days then added methanogenic microflora. PM represents the cultivations with raw coal and mixed culture of PF and methanogenic microflora. MM represents the cultivations with raw coal and methanogenic microflora. The 7 and 35 are incubation time.

The compositions of organic matter in the liquid phase in each sample are shown in Figure 4 and the details are presented in Supplementary Table 4. In the early stage of methane production, volatile fatty acids (VFAs), which are generally assumed to be important intermediates in the methanogenesis from coal (Yu et al., 2014), were the most abundant organic compounds. They accounted for 59.91, 57.70, and 36.48% in PP7, PM7, and MM7, respectively. This was distinct from the organic composition of the liquid obtained by soaking coal in anaerobic medium for 7 days (S7 in Figure 4). This was dominated by esters (41.79%), VFAs (23.93%), phenols (10.96%), and ketones (8.77%). This composition suggests that the changes of organic matter composition during methane production were mainly caused by functional microorganisms.

Compared with MM7, accumulations of VFAs in PP7 and PM7 were 64.21 and 58.15% higher. This is likely the reason for the increased methane production as a positive correlation between the increase of low molecular weight VFAs and higher methane production has been proved previously (Robbins et al., 2016). The higher VFA contents in PP7 and PM7 might be related to the activities of *Aspergillus* and *Penicillium* as these also sharply increased at the same time and both were reported to degrade complex organics with the VFA product (Schroeder, 1951; McDaniel et al., 1993). Aromatic acids and aromatic hydrocarbons accounted for 16.63 and 6.13% in MM, respectively. The aromatic acid content in MM7 was mainly composed of benzeneacetic acid, which was 1.36 and 4.11 times higher than that in PP7 and PM7, respectively. The higher VFAs and lower aromatic acids in PP7 and PM7 indicated that PF strengthened the ability of the microflora to degrade coal and intermediate metabolites, which led to higher methane production in cultivations PP and PM. Furthermore, aromatic hydrocarbons were not detected in PP7, showing that PF could effectively ferment the aromatics in coal.

At the end of cultivation, VFAs had been exhausted in PP35, PM35, and MM35, while esters, phenols, and ketones became dominant. The accumulation of these refractory substances (e.g., phenols and ketones) might inhibit the methane production (Wang et al., 2017). Although the structures of microbial communities of MM35, PM35, and PP35 were similar (Figure 2), the addition of PF caused certain differences to the organic matters in PP35 and PM35 from MM35. For example, the contents of esters in PP35 and PM35 were 36.95 and 29.99% lower than in MM35. This might be because *Aspergillus* and *Debaryomyces, two* ester-degraders, were more dominant in PP35 and PM35 (Besancon et al., 1995; Singh and Mukhopadhyay, 2012). The higher methane production in two treatments PP and PM would thus be related to the generation of the ingredients (VFAs and esters) of coal samples (Chen et al., 2017).

### Effect of Polycyclic Aromatic Hydrocarbons Degrading Fungal Flora on the Functional Groups in Coal During Methane Production

The ^13^C-NMR spectra of coal can be divided into three regions: aliphatic parts (14–90 ppm), aromatic parts (100–165 ppm), and carbonyl parts (165–220 ppm) (Li et al., 2015). The assignments and proportions of different functional groups in each sample are shown in Table 1. The proportions of aliphatic and aromatic carbons were similar across all samples and together accounted for more than 80%. Oxy-aliphatic carbons and methylene carbons were the main functional groups in aliphatic carbons. Protonated aromatic carbons accounted for more than half of all aromatic carbons. Two functional groups were detected in the group of carbonyl carbons including carboxyl carbons and carbonyl carbons.

**Table 1:** Assignments and percentages of different functional groups in coal based on ^13^C-NMR spectra.

**Peak**	**Chemical shift (ppm)**	**Carbon type**	**Relative area (%)**				
			**Raw coal**	**PP7**	**MM35**	**PP35**	**PM35**
**Aliphatic**						
1	14–16	Aliphatic CH_3_	1.30	1.00	1.10	0.91	1.12
2	16–22	Aromatic CH_3_	4.25	3.40	4.31	4.16	4.25
3	22–36	Methylene carbons	12.32	11.49	12.87	13.27	12.46
4	36–50	Methine and quaternary carbons	6.93	7.69	7.79	7.54	8.76
5	50–90	Oxy-aliphatic carbons	16.06	17.28	17.80	17.11	19.12
Sum			40.86	40.86	43.87	42.99	45.71
**Aromatic**						
6	100–129	Protonated aromatic carbons	23.61	18.88	21.22	22.8	22.72
7	129–137	Aromatic bridgehead carbons	5.63	6.19	4.89	5.29	6.14
8	137–148	Aromatic branched carbons	6.24	6.59	5.64	5.29	6.15
9	148–165	Oxy-aromatic carbons	8.36	8.89	8.42	8.09	5.87
Sum			43.84	40.55	40.17	41.47	40.88
**Carbonyl**						
10	165–180	Carboxyl carbons	9.53	10.49	9.15	9.32	6.55
11	180–220	Carbonyl carbons	5.77	8.09	6.82	6.23	6.85
Sum			15.30	18.58	15.97	15.55	13.40

After PF pretreatment for 7 days, both the protonated aromatic carbons and aromatic CH_3_ in PP7 decreased by about 20%, while carboxyl carbons and carbonyl carbons increased by 10.07 and 40.20%, respectively. This is consistent with the anaerobic degradation pathway of aromatic compounds, where aromatics are usually degraded by hydroxylation, carboxylation, and methylation. These degradations are performed by the extracellular enzymes secreted by fungi, such as lignin peroxidase, manganese peroxidase, ligninolytic enzymes, and laccase. Under the actions of *Acremonium, Penicillium*, and *Aspergillus* in PF, all of which could perform the above functions (Hao et al., 2010; Nedra et al., 2018), protonated aromatic carbons and aromatic CH_3_ in coal were gradually fermented to carboxyl and carbonyl carbons. Eventually, these were converted into H_2_, CO_2_, and VFAs for methane production (Strapoć et al., 2011; Nzila, 2018; Xia et al., 2020). Considering that C_2_H_4_O_2_, C_3_H_6_O_2_, C_4_H_8_O_2_, C_4_H_8_O_2_, and C_5_H_10_O_2_ were identified as the main intermediates in the initial stage of anaerobic biodegradation by PF (Supplementary Table 4), carboxylation was more likely to be the key reaction for the anaerobic metabolism of aromatics.

The increases of carboxyl carbons and carbonyl carbons caused by PF pretreatment were beneficial to the following biodegradation as oxygen-containing functional groups are the main target of microbial degradation in coal (Guo et al., 2021). By the coal structure at the end of PP cultivation, it could be further verified that carboxyl and carbonyl carbons in PP35 reduced by 11.15 and 22.99% comparing with PP7, respectively. Other oxygen-containing carbons decreased, including oxy-aromatic carbons and oxy-aliphatic carbons. The side chain of aromatics, aliphatic chains were also broken by microorganisms. Aromatic branched carbons and aromatic bridgehead carbons decreased by 19.73 and 14.54%, respectively, while aliphatic CH_3_, methine, and quaternary carbons decreased slightly, as about 9 and 2%, respectively. This indicates that the combination of PF and MM enhanced the bio-utilization of aromatics in coal.

In PM, carbonyl and oxygen-containing functional groups were also mainly affected by PF addition. The contents of carboxyl carbons and oxy-aromatic carbons in PM35 were 28.42 and 30.28%, respectively, lower than those in MM35 (Table 1). This suggests that the mixed culture of PF and MM improved the ability of microorganisms to degrade carbonyl and aromatic functional groups in coal, which contributed to the increased methane production.

## Conclusion

In this study, MM and PF were enriched. Two strategies for enhancing methane production by PF were explored including PP and PM. The results showed that both treatments increased methane production from coal. Both treatments altered the fungal community in the early stage of methane production, significantly increasing the proportion of *Penicillium* within 7 days of cultivation. These improved fungal communities promoted the accumulation of VFAs and the degradation of protonated aromatic carbon, aromatic CH_3_, and oxy-aromatic carbons in coal. These results suggest that methane production from lignite can be increased by both treatments using an indigenous PAH-degrading fungal flora. This provides a feasible way to enhance biomethane generation in coal seams. Considering the complexity and elapsed time of procedure, the method of PM is more convenient for on-site operation that it would not change the implementation of microflora injection and methane extraction which is the main procedure of MECBM (Ritter et al., 2015).

## Data Availability Statement

The original contributions presented in the study are included in the article/Supplementary Material, further inquiries can be directed to the corresponding author/s. The 16S rRNA gene and ITS gene sequences derived from Miseq were deposited in the NCBI Sequence Read Archive under accession number PRJNA754200.

## Author Contributions

KG and YZ: writing - original draft. KG, HG, ZH, MU, and MA: writing - review and editing. YZ: investigation. HG: conceptualization, funding acquisition, and supervision. All authors contributed to the article and approved the submitted version.

## Funding

This work was supported by the National Natural Science Foundation of China (U1810103 and 51404163), Key R&D program of Shanxi Province (International Cooperation, 201903D421088), and Coal Seam Gas Joint Foundation of Shanxi (2014012006).

## Conflict of Interest

The authors declare that the research was conducted in the absence of any commercial or financial relationships that could be construed as a potential conflict of interest.

## Publisher's Note

All claims expressed in this article are solely those of the authors and do not necessarily represent those of their affiliated organizations, or those of the publisher, the editors and the reviewers. Any product that may be evaluated in this article, or claim that may be made by its manufacturer, is not guaranteed or endorsed by the publisher.
