# Supplementary material for: Enhancing Biomethane Production From Lignite by an Anaerobic Polycyclic Aromatic Hydrocarbon Degrading Fungal Flora Enriched From Produced Water
Source: Front Microbiol. 2022 May 26;13:899863. doi: 10.3389/fmicb.2022.899863 (PMC9197214; doi:10.3389/fmicb.2022.899863)
Supplement: Supplementary file 1 [file Data_Sheet_1.PDF]

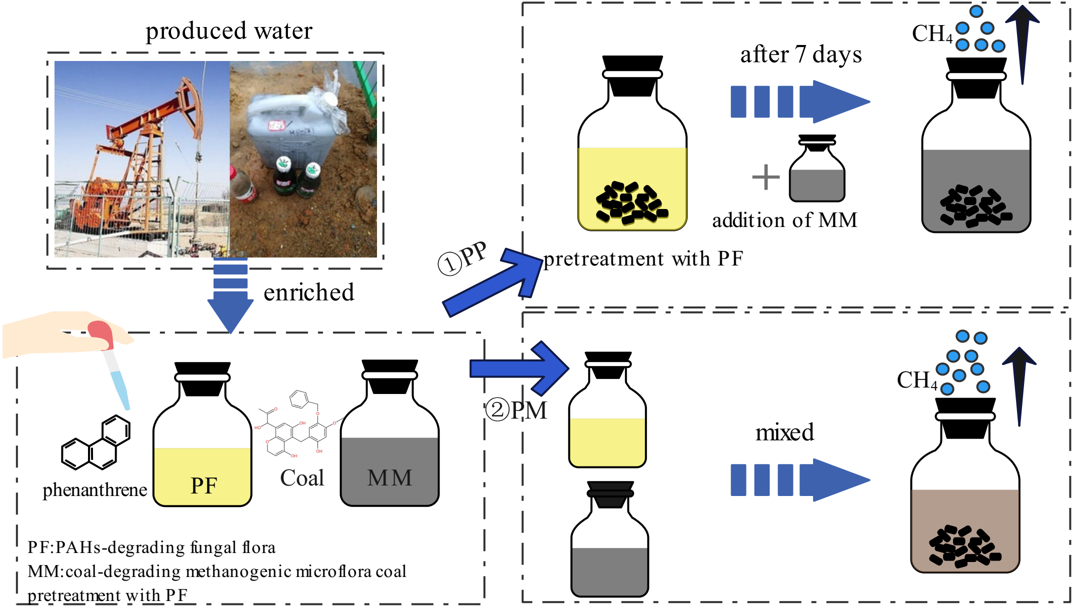
produced water
enriched
phenanthrene
PF
Coal
MM
PF:PAHs-degrading fungal flora
MM:coal-degrading methanogenic microflora coal
pretreatment with PF
①PP
pretreatment with PF
after 7 days
addition of MM
CH_4
②PM
mixed
CH_4
