# Supplementary material for: Enhancing Biomethane Production From Lignite by an Anaerobic Polycyclic Aromatic Hydrocarbon Degrading Fungal Flora Enriched From Produced Water
Source: Front Microbiol. 2022 May 26;13:899863. doi: 10.3389/fmicb.2022.899863 (PMC9197214; doi:10.3389/fmicb.2022.899863)
Supplement: Supplementary file 2 [file Data_Sheet_2.pdf]

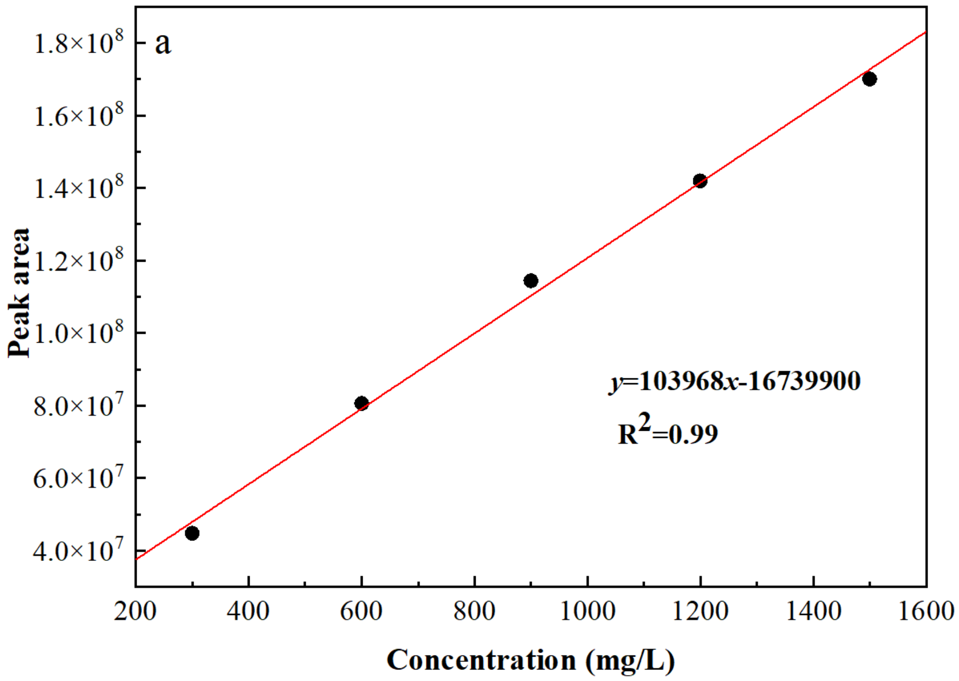

Figure S1 Standard curves of phenanthrene concentration determined by GC-MS.

Table S1 Summary of microbial diversity estimators

	Samples	Reads	OTUs	Chao1	Shannon	Simpson	Coverage
Fungi	PF	38377	21	21	2.43	0.1306	1
	MM	40560	198	201	3.99	0.0367	0.999870
	MM7	59107	100	100	3.28	0.0812	0.999983
	MM35	37308	161	164	4.06	0.0356	0.999920
	PP7	26636	63	63	1.93	0.2723	0.999887
	PP35	39839	176	179	4	0.0377	0.999900
	PM7	23352	71	72	1.81	0.3828	0.999872
	PM35	40274	173	181	3.94	0.0372	0.999851
Archaea	MM	29827	97	126	0.82	0.7356	0.999363
	MM7	59107	100	100	3.28	0.0812	0.999983
	MM35	35615	23	23	0.57	0.7606	0.999944
	PP35	60808	26	27	0.7	0.6755	0.999967
	PM7	23691	30	31	1.78	0.2487	0.999916
	PM35	63063	24	25	0.41	0.8352	0.999952

Table S2 Fungal communities at the genus level in each sample

Genus	PF	MM	MM7	PP7	PM7	MM35	PP35	PM35
Acremonium	14.79	1.12	1.04	0.5	0.62	1.79	1.69	1.57
Alternaria	1.37	7.1	2.06	0.28	0.92	6.21	3.15	4.35
Aspergillus	9.1	7.74	14.93	22.08	11.84	6.44	9.43	13.13
Aureobasidium	0	0.03	1.28	0	0	0.02	0	0
Beauveria	0	0.28	0	0	0	1.29	0	0.21
Candida	8.2	1.12	1.22	0.15	0.27	1.9	1.17	1.93
Chaetomium	0	1.1	2.82	0.06	0.22	1.3	0.41	0.66
Cladosporium	2.75	4.88	3.43	1.09	0.15	2.84	3.99	2.74
Colletotrichum	0	1.05	0.7	0	0	0.78	1.92	2.84
Coniothyrium	0	2.55	0	0	0.03	1.17	1.02	2.61
Coprinopsis	0	1.17	0	0	0	0.3	1.16	0
Debaryomyces	6.93	2.31	10.85	0.01	0	1.3	7.51	1.68
Dicyma	0	0.06	1.52	1.65	1.78	0.22	0.08	0.17
Eupenidiella	0	0	2.11	0	0	0	0	0
Filobasidium	1.9	0.4	0.61	0	0.36	0.65	0	0.2
Fusarium	4.83	14.24	1.61	1.26	0.76	11.97	8.73	12.56
Ganoderma	0	0.5	0	0	0	1.84	2.74	1.88
Humicola	3.45	1.7	0.39	0.21	0	0.87	1.09	1.24
Malassezia	0	0.08	1.99	1.72	0.42	0	0.23	0.35
Mortierella	0	0.96	0.48	0	0.01	1.4	1.49	1.4
Naganishia	0	11.42	0.75	0	0.28	9.61	14.22	13.95
Neosartorya	0	0.83	1.41	0.21	1.64	2.91	0.92	0.86
Parengyodontium	4.15	1.67	0.29	0	0.19	0.87	0.79	1.25
Penicillium	8.89	13.04	41.1	68.79	78.01	17.74	13.62	12.42
Podospora	1.76	0.2	0.21	0	0	0	0	0

Rhodotorula	0	1.54	0.49	0.23	0.05	0.99	2.63	3.12
Sarocladium	0	0.92	0.11	0	0	1.14	0.74	0.27
Sclerotinia	0	0.58	0	0	0	0.24	0	1.2
Simplicillium	1.06	0	0	0	0.12	0	0.26	0.12
Talaromyces	27.87	0	0.32	0.75	0.25	0.53	0.52	0.71
Trichoderma	0	6.15	0.32	0.18	0.54	4.03	1.57	3.15
Vishniacozyma	0	0	0	0	0.2	0.12	0.29	1.24
Unclassified	1.62	2.47	1.68	0.16	0.35	5.09	3.71	2.2
Others	1.32	12.77	6.28	0.68	0.98	14.48	14.88	10

0% 50% 100%

Note: The genera accounted for < 1 % of sequence reads in each sample were grouped into others.

Table S3 The archaeal communities at the genus level in each sample

Genus	MM	MM7	MM35	PM7	PM35	PP35
Methanosarcina	95.17	81.24	88.11	25.46	92.38	82.49
Bathyarchaeia_norank	4.54	4.89	10.80	74.53	7.39	17.17
Methanolobus	0.10	0.04	0.84	0.00	0.15	0.06
Methanobrevibacter	0.00	0.73	0.00	0.02	0.00	0.00
Candidatus Nitrosopumilus	0.00	0.00	0.00	0.00	0.00	0.00
Candidatus Methanofastidiosum	0.02	0.00	0.01	0.00	0.00	0.00
Unclassified	0.17	13.09	0.25	0.00	0.07	0.29

0% 50% 100%

Note: The genera accounted for < 1 % of sequence reads in each sample were grouped into others.

Table S4 Distribution of organic matters in each sample

Samples	Retention time (min)	Compound names	Molecular formula	Percentage (%)
S7	7.435	Acetic acid	$C_2H_4O_2$	4.38
	8.079	Propanoic acid	$C_3H_6O_2$	2.43
	8.302	Propanoic acid, 2-methyl-	$C_4H_8O_2$	0.80
	8.784	Butanoic acid	$C_4H_8O_2$	10.98
	9.147	Butanoic acid, 2-methyl-	$C_5H_{10}O_2$	5.34
	10.284	Propanoic acid, 2-chloro-, 2-propenyl ester	$C_6H_9ClO_2$	0.54
	11.7	Butylated Hydroxytoluene	$C_{15}H_{24}O$	1.41
	13.039	Phenol	C_6H_6O	6.51
	17.418	2,4-Di-tert-butylphenol	$C_{14}H_{22}O$	3.04
	20.147	5-Hydroxymethyldihydrofuran-2-one	$C_5H_8O_3$	1.64
	20.874	1,2-Benzenedicarboxylic acid, bis(2-methylpropyl) ester	$C_{16}H_{22}O_4$	2.04
	21.294	Benzeneacetic acid	$C_8H_8O_2$	0.98

	21.963	2(3H)-Furanone, dihydro-4-hydroxy-	$C_4H_6O_3$	1.89
	23.188	Dibutyl phthalate	$C_{16}H_{22}O_4$	2.91
	23.527	o-Acetyl-L-serine	$C_5H_9NO_4$	9.20
	23.857	7,9-Di-tert-butyl-1-oxaspiro (4,5) deca-6,9-diene-2,8-dione	$C_{17}H_{24}O_3$	5.24
	25.144	Bis(2-ethylhexyl) phthalate	$C_{24}H_{38}O_4$	36.30
	26.887	n-Hexadecanoic acid	$C_{16}H_{32}O_2$	4.37
PP7	7.414	Acetic acid	$C_2H_4O_2$	6.57
	8.063	Propanoic acid	$C_3H_6O_2$	9.84
	8.291	Propanoic acid, 2-methyl-	$C_4H_8O_2$	4.89
	8.774	Butanoic acid	$C_4H_8O_2$	13.98
	9.142	Butanoic acid, 2-methyl-	$C_5H_{10}O_2$	16.66
	9.786	Pentanoic acid	$C_5H_{10}O_2$	0.78
	10.455	Pentanoic acid, 4-methyl-	$C_6H_{12}O_2$	12.08
	11.7	Butylated Hydroxytoluene	$C_{15}H_{24}O$	1.43
	13.039	Phenol	C_6H_6O	8.10
	17.415	2,4-Di-tert-butylphenol	$C_{14}H_{22}O$	1.99

	18.58	DL-Norleucine	$C_6H_{13}NO_2$	0.71
	19.94	Cyclopropanetetradecanoic acid, 2-octyl-, methyl ester	$C_{26}H_{50}O_2$	1.36
	20.147	5-Hydroxymethyldihydrofuran-2-one	$C_5H_8O_3$	3.02
	21.284	Benzeneacetic acid	$C_8H_8O_2$	3.87
	21.969	2(3H)-Furanone, dihydro-4-hydroxy-	$C_4H_6O_3$	1.52
	22.226	Hydrocinnamic acid	$C_9H_{10}O_2$	8.30
	23.183	1,2-Benzenedicarboxylic acid, butyl octyl ester	$C_{20}H_{30}O_4$	0.44
	23.52	Aziridine-2-carbothioamide	$C_3H_6N_2S$	3.02
	26.882	n-Hexadecanoic acid	$C_{16}H_{32}O_2$	1.44
PM7	4.742	p-Xylene	C_8H_{10}	2.46
	4.825	p-Xylene	C_8H_{10}	1.51
	4.882	p-Xylene	C_8H_{10}	1.40
	6.091	3-Hexen-1-ol, (E)-	$C_6H_{12}O$	1.08
	7.43	Acetic acid	$C_2H_4O_2$	2.31
	8.047	Propanoic acid	$C_3H_6O_2$	17.41
	8.286	Propanoic acid, 2-methyl-	$C_4H_8O_2$	1.28

8.421	Hexadecane	$C_{16}H_{34}$	0.74
8.779	Butanoic acid	$C_4H_8O_2$	1.60
9.127	Butanoic acid, 3-methyl-	$C_5H_{10}O_2$	16.90
10.439	Pentanoic acid, 4-methyl-	$C_6H_{12}O_2$	18.20
11.695	Butylated Hydroxytoluene	$C_{15}H_{24}O$	0.71
13.023	Phenol	C_6H_6O	5.65
14.071	p-Cresol	C_7H_8O	3.92
19.888	2-t-Butyl-5-propyl-[1,3]dioxolan-4-one	$C_{10}H_{18}O_3$	2.14
20.858	1,2-Benzenedicarboxylic acid, bis(2-methylpropyl) ester	$C_{16}H_{22}O_4$	2.93
21.247	Benzeneacetic acid	$C_8H_8O_2$	4.05
22.726	Cyclopropanetetradecanoic acid, 2-octyl-, methyl ester	$C_{26}H_{50}O_2$	0.91
22.954	Octadecanoic acid	$C_{18}H_{36}O_2$	1.22
23.172	Dibutyl phthalate	$C_{16}H_{22}O_4$	3.52
23.536	Aziridine-2-carbothioamide	$C_3H_6N_2S$	3.59
23.831	7,9-Di-tert-butyl-1-oxaspiro (4,5) deca-6,9-diene-2,8-dione	$C_{17}H_{24}O_3$	3.37
25.211	Pyrrolo [1,2-a] pyrazine-1,4-dione, hexahydro-3-(2-methylpropyl)-	$C_{11}H_{18}N_2O_2$	0.94

	26.846	n-Hexadecanoic acid	$C_{16}H_{32}O_2$	2.20
MM7	4.732	o-Xylene	C_8H_{10}	4.05
	4.81	p-Xylene	C_8H_{10}	2.09
	7.415	Acetic acid	$C_2H_4O_2$	6.75
	8.058	Propanoic acid	$C_3H_6O_2$	11.14
	8.281	Propanoic acid, 2-methyl-	$C_4H_8O_2$	5.59
	8.421	Hexadecane	$C_{16}H_{34}$	0.52
	8.769	Butanoic acid	$C_4H_8O_2$	13.01
	9.132	Hexanoic acid, 2-methyl-	$C_7H_{14}O_2$	21.52
	10.279	Propanoic acid, 2-chloro-, 2-propenyl ester	$C_6H_9ClO_2$	0.49
	11.7	Butylated Hydroxytoluene	$C_{15}H_{24}O$	3.29
	13.023	Phenol	C_6H_6O	2.28
	14.077	p-Cresol	C_7H_8O	2.13
	18.565	1-Hexadecanol, 2-methyl-	$C_{17}H_{36}O$	0.56
	20.864	1,2-Benzenedicarboxylic acid, bis(2-methylpropyl) ester	$C_{16}H_{22}O_4$	2.04
	21.247	Benzeneacetic acid	$C_8H_8O_2$	16.63

	23.172	Dibutyl phthalate	$C_{16}H_{22}O_4$	2.54
	23.837	7,9-Di-tert-butyl-1-oxaspiro (4,5) deca-6,9-diene-2,8-dione	$C_{17}H_{24}O_3$	2.18
	26.851	n-Hexadecanoic acid	$C_{16}H_{32}O_2$	3.22
PP35	11.695	Butylated Hydroxytoluene	$C_{15}H_{24}O$	4.12
	13.044	Phenol	C_6H_6O	17.36
	14.813	2-Piperidinone	C_5H_9NO	1.54
	17.418	2,4-Di-tert-butylphenol	$C_{14}H_{22}O$	22.51
	19.94	Cyclopropanetetradecanoic acid, 2-octyl-, methyl ester	$C_{26}H_{50}O_2$	4.04
	20.153	5-Hydroxymethyldihydrofuran-2-one	$C_5H_8O_3$	13.09
	21.979	2(3H)-Furanone, dihydro-4-hydroxy-	$C_4H_6O_3$	7.38
	22.809	Octadecanoic acid, 4-hydroxybutyl ester	$C_{22}H_{44}O_3$	1.56
	22.991	Cyclopropanetetradecanoic acid, 2-octyl-, methyl ester	$C_{26}H_{50}O_2$	4.36
	23.178	1,2-Benzenedicarboxylic acid, butyl octyl ester	$C_{20}H_{30}O_4$	2.09
	23.544	Aziridine-2-carbothioamide	$C_3H_6N_2S$	10.57
	26.877	n-Hexadecanoic acid	$C_{16}H_{32}O_2$	11.37
PM35	5.884	Styrene	C_8H_8	3.20

	6.776	2-Cyclopenten-1-one	C_5H_6O	3.05
	9.64	2-Penten-1-ol, (E)-	$C_5H_{10}O$	5.35
	10.284	Propanoic acid, 2-chloro-, 2-propenyl ester	$C_6H_9ClO_2$	2.12
	11.7	Butylated Hydroxytoluene	$C_{15}H_{24}O$	5.71
	13.036	Phenol	C_6H_6O	35.55
	14.087	p-Cresol	C_7H_8O	7.69
	15.586	Caprolactam	$C_6H_{11}NO$	3.17
	20.868	1,2-Benzenedicarboxylic acid, bis(2-methylpropyl) ester	$C_{16}H_{22}O_4$	7.99
	23.177	Dibutyl phthalate	$C_{16}H_{22}O_4$	8.91
	23.847	7,9-Di-tert-butyl-1-oxaspiro(4,5)deca-6,9-diene-2,8-dione	$C_{17}H_{24}O_3$	12.47
	26.872	n-Hexadecanoic acid	$C_{16}H_{32}O_2$	4.78
MM35	6.543	2-Pentenal, (E)-	C_5H_8O	2.59
	6.781	2-Cyclopenten-1-one	C_5H_6O	4.43
	9.64	2-Penten-1-ol, (E)-	$C_5H_{10}O$	5.17
	10.45	Pentanoic acid, 4-methyl-	$C_6H_{12}O_2$	2.74
	13.034	Phenol	C_6H_6O	12.58

14.087	p-Cresol	C_7H_8O	2.56
15.581	Caprolactam	$C_6H_{11}NO$	1.72
15.815	Diethyl azelate	$C_{13}H_{24}O_4$	1.37
16.38	Hexadecanoic acid, ethyl ester	$C_{18}H_{36}O_2$	9.12
17.403	2,4-Di-tert-butylphenol	$C_{14}H_{22}O$	8.40
19.006	Dodecanedioic acid, dimethyl ester	$C_{14}H_{26}O_4$	2.72
19.847	(E)-9-Octadecenoic acid ethyl ester	$C_{20}H_{38}O_2$	12.66
20.594	Ethyl 9.cis.,11. trans. -octadecadienoate	$C_{20}H_{36}O_2$	3.72
20.869	1,2-Benzenedicarboxylic acid, bis(2-methylpropyl) ester	$C_{16}H_{22}O_4$	6.35
22.498	Linoleic acid ethyl ester	$C_{20}H_{36}O_2$	2.96
23.188	Dibutyl phthalate	$C_{16}H_{22}O_4$	8.05
23.847	7,9-Di-tert-butyl-1-oxaspiro (4,5) deca-6,9-diene-2,8-dione	$C_{17}H_{24}O_3$	4.51
26.384	Ethyl Oleate	$C_{20}H_{38}O_2$	2.05
26.877	n-Hexadecanoic acid	$C_{16}H_{32}O_2$	6.30
